# Human Temperaments. X.—The Jealous Temperament


**Published:** 1916-09-09

**Authors:** 


### HUMAN TEMPERAMENTS.

### X.—The Jealous Temperament.*

The difference between jealousy and envy was outlined in the last paper. Envy is aroused by the contemplation of but one other person: jealousy depends on the relation between two other persons, and it is curious that although jealousy is a very widespread passion, to which everyone is liable in some degree, a passion which affects not only mankind, but many of the lower animals also, and frequently produces disastrous consequences, yet the double attitude of the jealous person has never become embodied in language. A man is said to be "jealous of" the other man to whom his girl shows an inclination, but there is no phrase to express the corresponding feeling that he entertains towards the girl.

### Jealousy and the Sense of Proprietorship.

The type of jealousy is sexual jealousy, but jealousy is by no means exclusively sexual. The sycophant is jealous of the person to whom his patron shows inclination. The admiring schoolgirl is jealous of the other girl to whom her adored mistress shows inclination. The child that passionately loves its mother is jealous of the new baby on which the mother lavishes caresses. The basis of jealousy is the desire for the exclusive possession of the love, interest, or attention of another person, which the jealous person desires to hold in monopoly, and jealousy arises when this monopoly is infringed. Of the love, the interest, the concern, the attention of another person the jealous regards himself as the proprietor, and his jealousy is excited when his proprietary rights are infringed. Thus jealousy is but a special case of a much more general passion. The type of the jealous person commonly accepted is the man whose girl prefers another man, but a more general type is the dog snarling over a bone. The dog regards the bone as his own property, to which he has the right of exclusive possession, and he is ready to defend his right, and to attack anyone who presumes to infringe it; and he proclaims this readiness by his snarl, which is a threat of retaliation upon anyone who presumes to interfere with his proprietary right. So the man feels that the affection, regard, concern, and attention of his girl are his own property, to which he has the right of exclusive possession, and he is ready to assert his right and attack anyone who presumes to interfere with it. The man of jealous temperament is he who has his sense of proprietorship strongly developed, and is sensitive to any interference with it—nay, to any approach to interference, to any possibility or presumption of interference. The jealous dog does not wait until an attempt is made to rob him of his bone; he snarls at the passer-by who is not thinking of him; and the jealous man does not wait to feel jealousy until his exclusive possession is actually interfered with: the mere approach to interference, the possibility of interference, is enough to excite his jealousy.

Jealousy, then, is the feeling that is aroused in us by interference with our proprietorship. It is usually taken as limited to interference with our proprietorship in the affection and regard of a person of the opposite sex, but the feeling thus aroused is indistinguishable from that aroused by diverting the affection and regard of anyone, even of the same sex, and is not widely different from the feeling aroused by interference with any other mode of proprietorship. To use a man's horses or motor-car, or his camera or cricket-bat, or to smoke his cigars or drink his wines without his permission, arouses in him an emotion which is scarcely distinguishable from jealousy.

Jealousy, therefore, is by no means dependent on love. It is often associated with love, but it is not necessarily so. Even sexual jealousy may be felt without any intermixture of love. It is by no means unusual for a woman who does not care a straw for her husband, who may even detest him, to be frantically jealous of his attentions to other women. It is not the outraged love that provokes jealousy; it is the infringement of proprietorship. Little as she cares for her husband, she has a strong sense of her proprietorship not over him, but over his attentions, and if they are directed elsewhere her jealousy is aroused.

### The Provocation to Retaliation.

Nor is jealousy by any means exclusively directed towards persons of the sex of the jealous, or towards persons at all. A husband of jealous temperament will be jealous of the man towards whom his wife seems to incline, but he will also be jealous of her woman friend, of her and his own children, even of her lap-dog or her cat. A jealous wife will be jealous not only of her husband's women friends, but also of anything that engrosses a share of that attention to which she lays exclusive claim. She will be jealous of his men friends, of his yacht, his golf, his books, his gun, even of his business, for all of these steal from her a portion of that attention that she seeks solely to engross.

The natural manifestation of envy is, as we have seen in a previous article, retaliation against the envied person, but is by no means restricted to this mode of expression. If such retaliation is impracticable, or even in addition to it, the envied vents his spleen on innocent third parties, or perhaps by a strange distortion of purpose punishes himself. The expression of jealousy is no less diffused and multiplied. It provokes to retaliation, and the retaliation may be directed against either the person or thing towards which the jealousy is felt, or against the person or thing on account of which

* The previous articles appeared on May 6, 13, 27, June 10 and 24, July 8 and 22, August 5 and 19, 1916; and a letter on the subject on May 20.

the jealousy is felt, or against the jealous person himself, or, though more rarely, against an innocent fourth party.

The most natural manifestation of jealousy is retaliation against the intruder—against the person who has infringed the proprietorship of the jealous person, or what he conceives to be his proprietorship—a conception often formed on very inadequate grounds. The person towards whom the jealousy is felt may have taken no active step against the proprietorship, may be ignorant that it has been infringed, may be ignorant that there is any proprietorship, or that any is claimed. A woman may be furiously jealous of the woman to whom her husband or her lover appears to be attracted, even though no such attraction is in fact felt, even though the other woman is ignorant of it if it is felt. A ludicrous instance of jealousy has recently come under the notice of the writer, an instance in which a man conceived a furious jealousy of another who had written upon a subject which the jealous man regarded as his own property; and this although the intruder was totally ignorant that the jealous man had written on that subject, and although others had written on it before the jealous man had done so. But however groundless the jealousy, however absurd the grounds that give rise to it, it is one of the most lethal of the passions, and more often than any other passion prompts to acts of deadly animosity. Although, however, the person towards whom the jealousy is directed is an object of this animosity, he is not the sole, nor even usually the chief object. In sexual jealousy the retaliation is more often directed upon the person on account of whom the jealousy is felt than towards the person of whom the jealousy is felt. Of the hundreds of assaults committed on the prompting of jealousy that come before the courts every year, but few are committed upon the rival, and these much more often by the jealous woman than by the jealous man. The jealous man does not as a rule assault his rival; he beats his wife or his girl, or cuts her throat. The jealous woman does not as a rule assault her husband or her lover. She seeks out her rival and scratches her face or perhaps throws vitriol over her.

### A Strange Vagary.

What would be very surprising if it were not so familiar is that jealousy so frequently prompts the jealous man to injure neither his rival nor the woman who has given, as he thinks, occasion for jealousy, but himself. That he should try to get his rival out of his way is intelligible; that he should punish the woman who has transferred her allegiance is intelligible; but that he should punish himself, often with capital punishment, is indeed a strange vagary. When he has murdered his faithless wife or lover, it is perhaps not unnatural that he should kill himself, both to forestall the punishment of the law and because he has now deprived himself of that which made his life most worth living; but that he, or more often she, should kill herself for the motive of jealousy is less explicable. It can be explained, however, by the consideration that has just been mentioned. The deprivation of her lover deprives the girl, for the time being, of her whole motive and purpose in life. Life is to every man, and still more to every woman, not a gift, but a trust, to be handed on to a succeeding generation. Unknown to herself, unrealised by herself, the craving for motherhood dominates her being. As long as the future has in store for her the realisation and satisfaction of this craving, even if only in possibility, she can endure in hope and tranquillity; but when by the securing of a lover she has been brought within sight of the realisation of her hope, and when by his desertion the cup is dashed from her lips, all interest in life is abolished, and she hastens to quit the scene that is become distasteful to her.

Jealousy, in some degree, is natural to man and woman, and all possess it more or less; but in the jealous temperament it assumes a dominant influence. The man of jealous temperament is furious if his wife but shows interest in the conversation of her partner at a dinner; the woman of jealous temperament is furious if her husband is even commonly polite to a lady guest. Persons of this temperament may be quite aware of the groundlessness and absurdity of their jealousy. They may deplore it and despise themselves for it; but for all that it dominates them, and they cannot help it.

